# Antibiotic resistance characteristics and risk factors analysis of *Helicobacter pylori* strains isolated from patients in Liaoning Province, an area in North China

**DOI:** 10.7717/peerj.15268

**Published:** 2023-05-16

**Authors:** Yanmeng Wang, Yiling Li, Yuehua Gong, Yuzhen Dong, Jing Sun, Moye Chen

**Affiliations:** 1 Gastroenterology, The First Hospital of China Medical University, Shenyang, Liaoning, China; 2 Key Laboratory of GI Cancer Etiology and Prevention, The First Hospital of China Medical University, Shenyang, Liaoning, China

**Keywords:** *Helicobacter pylori*, Antibiotic resistance, Eradication, Multi-resistance, Regional characteristic, Liaoning Province

## Abstract

**Background:**

The prevalence of *Helicobacter pylori* (*H. pylori*) keeps rising while the eradication rate continues to decline due to the increasing antibiotic resistance. Regional variations of antimicrobial resistance to *H. pylori* have been recommended by guidelines in recent years. This study aims to investigate the antibiotic resistance rate of *H. pylori* and its association with infected subjects’ characteristics in Liaoning Province, an area in north China.

**Methods:**

Gastric tissues from 178 *H. pylori* positive participants without previous antibiotic use within four weeks were collected for *H. pylori* culture. Antibiotic susceptibility to furazolidone (AOZ), tetracycline (TC), levofloxacin (LFX), metronidazole (MET), clarithromycin (CLA), and amoxicillin (AMX) were examined with the agar dilution method. Associations between *H. pylori* resistance and patient characteristics were further analysed.

**Results:**

No resistance was observed in AOZ or TC. For LFX, MET, CLA, and AMX, the overall resistance rates were 41.10%, 79.14%, 71.78%, and 22.09% respectively. There were significant differences between resistance to CLA and MALToma (*P* = 0.021), and between resistance to MET and age (*P* < 0.001).

**Conclusions:**

The primary resistant rates of LEX, MET, CLA, and AMX were relatively high in Liaoning. Treatment effectiveness improvement could be achieved by prior antimicrobial susceptibility tests before antibiotic prescription.

## Introduction

*Helicobacter pylori* (*H. pylori*) is a ubiquitous bacterium which is well resistant to the gastric acid. It was estimated that the global prevalence of *H. pylori* was approximately 50% (Hooi et al., 2017). Previous studies established that the colonization of *H. pylori* was directly involved in the occurrence of various gastrointestinal diseases (Kishikawa et al., 2020; Yamaoka, 2018). Emerging evidence has suggested that *H. pylori* eradication could cure gastrointestinal lesions (Lanas & Chan, 2017; Sonnenberg, Turner & Genta, 2020) , delay the development of gastric cancer (Kosunen et al., 2011) , and reducing the recurrence of peptic ulcers and gastric cancers (Chey et al., 2017; Malfertheiner et al., 2017). Thus, it is essential to conduct eradication treatment of this bacteria.

Currently there are three main antibiotic-based eradication strategies of *H. pylori* infection: bismuth quadruple, triple therapy and sequential therapy (Chey et al., 2017). *H. pylori* treatment guidelines recommend different regimens in various countries. Cho & Jin (2022) discussed in detail recent guidelines for *H. pylori* treatment among China, Japan and South Korea, summarized the eradication rates of numerous clinical trials in each country. They revealed current situation of clinical regimen chosen in various countries. The most commonly selected antibiotics in China are furazolidone (AOZ), tetracycline (TC), levofloxacin (LFX), metronidazole (MET), clarithromycin (CLA), and amoxicillin (AMX). Unfortunately, the eradication rate of *H. pylori* has fallen due to ever increasing antibiotic resistance during the last decades. A systematic review and meta-analysis found the overall eradication rate of *H. pylori* resistant to antibiotics was significantly more than 16% lower than that sensitive to antibiotics (Zou et al., 2020). Scientists reported that primary resistance to LFX and MET respectively was more than 15% worldwide (Savoldi et al., 2018). Further analyses concordantly showed evident regional differences in antibiotic resistance to *H. pylori* (Megraud et al., 2021). For example, one study has shown that the antibiotic resistance to CLA was 31.1% in the Japanese population (Okamura et al., 2014). While in Portugal and in the southeast area of Vietnam, the rate of CLA-resistance was reported to be 42% and 72.6%, respectively (Dang et al., 2020; Lopo et al., 2018). Meanwhile, in China, a large country in Asia, the infection rate of *H. pylori* was almost 55.8%, and the antibiotic resistance differs greatly in various areas (Hooi et al., 2017). The resistance rate of MET was up to 95.5% in Jiaxing City, whereas in Beijing the rate was only 66.8% in the samples obtained during 2009 and 2010 (Ji et al., 2016; Zhang et al., 2015b). Till now, the *H. pylori* antibiotic resistance rates and patterns in Liaoning, an area of north China, remain unclear.

Risk factors for *H. pylori* antibiotic resistance have guiding significance in clinical practice, several studies were conducted to explore associations between related factors and their regional antibiotic resistance situation (Boyanova et al., 2012; Ji et al., 2016). By assessing whether infected patients have or how many factors they have, physicians would be instructed to evaluate possible antibiotic resistance. Therefore, analysing correlations between them in separated regions is of great significance to guide clinical drug use for physicians and prevent the occurrence of drug resistance for patients.

In the current study, we investigated the antibiotic resistance to AOZ, TC, LFX, MET, CLA, and AMX in Liaoning Province. Risk factors for antibiotic resistance were further evaluated. Our study intended to provide guidance for the individualized treatment of *H. pylori*, and thus to improve the eradication rate in Liaoning Province.

## Materials & Methods

### Selection of patients

The enrolled subjects were all patients at the gastroenterology department of the First Affiliated Hospital of China Medical University, a tertiary hospital in Liaoning Province during March 2021 to August 2022. Patients enrolled came from various cities in Liaoning Province. Every selected patient was *H. pylori* positive and has not received any antibiotic use in the last four weeks. Subjects positive for *H. pylori* infection, aged ≥18 years were recruited, with at least one of the following diseases: superficial gastritis (SG), atrophic gastritis (AG), gastric cancer (GC), mucosa associated lymphoid tissue lymphoma (MALToma) and peptic ulcer diseases (PUD). The exclusion criteria included subjects administered any antibiotics in the past 4 weeks.

### Acquisition of clinical specimens and Basic information

Gastric mucosa tissues of the subjects were obtained through the endoscopic biopsy process to confirm the infectious status of *H. pylori* and to perform the antibiotic sensitivity test. Basic information of the subjects was obtained from HIS Information System Technical Support Services, including age, gender, current smoking status, current drinking conditions, BMI, hypertension, diabetes mellitus, endoscopic diagnosis, and *H. pylori* treatment history. The definition of current smoking and drinking in the present study is that participated subjects engaged in smoking and/or drinking behaviour in accordance with their personal smoking and/or drinking habits during the first 4 weeks before conducting the drug sensitivity test. This project was approved by the Human Ethics Review Committee of the First Affiliated Hospital of China Medical University (2021325). We have received written informed consent from participants.

### Determination of *H. pylori* infection

Urea breath test (UBT) was performed with The Kit For ^13^C-Urea Breath Test (Haiderun Pharmaceutical Group Co. LTD., Beijing, China) through intaking 100-mg ^13^C-labelled reagent. The WLD600C^13^C Analyser (Haiderun Pharmaceutical Group Co. LTD., Beijing, China) was applied to analyse the breath samples and a positive result was decided following instructions of the manufacturer. Three gastric biopsy specimens of each subject (one from the antrum, one from the angle and the other from the corpus) were placed in a *H. pylori* transport culture medium (patent number: CN104762235A) (Guo, 2018) and transferred on ice to the *H. pylori* laboratory at Chain Medical Labs in Changchun, China (91220101MA0Y6MJX1T). Then the samples were fully grounded and inoculated into Columbia blood agar (CBA) plates, supplemented with 5% sheep blood. The colony of *H. pylori* was observed after being cultured on the plate under microaerobic conditions (5% oxygen, 85% nitrogen, and 10% carbon dioxide) at 37 °C for three to four days. Positive urease, catalase and oxidase tests and Gram-staining were performed to identify colonies resembling *H. pylori* (Blanchard & Nedrud, 2012). The patient who received positive results from both UBT and culture was identified to be *H. pylori*-positive.

### Test of antimicrobial susceptibility

The minimal inhibitory concentration (MIC) of *H. pylori* to six antibiotics (AOZ, TC, LFX, MET, CLA, and AMX) was measured by the agar dilution method. Various concentrations of the targeted antibiotics were diluted in the agar mediums (Hangzhou Haiji Biotechnology Co. LTD., Zhejiang, China). Suspensions of *H. pylori* strains were transferred onto the plates, then we incubated those plates at 37 °C under microaerophilic conditions (85% nitrogen, 10% carbon dioxide, and 5% oxygen). After 72-hour cultivation, the presence of *H. pylori* colonies was observed. ATCC43504 was selected as a quality control. Resistance breakpoints of AOZ, TC, LFX, MET, CLA, and AMX were defined as MIC > 1, >1, >1, >8, >0.5, and >0.125 µg/mL, respectively, in accordance with the guidance of the European Committee on Antimicrobial Susceptibility Testing (EUCAST) (https://eucast.org/clinical_breakpoints/).

### Statistical analyses

All statistical analyses were performed using the statistical software SPSS (version 18.0; SPSS Inc., Chicago, IL, United States). Categorical data were presented as number and percentage and continuous data were presented as mean  ± standard deviation (SD). The following characteristics data from participated subjects were processed using descriptive statistical analysis, including age, gender, current smoking status, current drinking conditions, BMI, hypertension, diabetes mellitus, endoscopic diagnosis, *H. pylori* treatment history, and antibiotic susceptibility. Chi-square test was applied to compare the difference between groups. Fisher’s exact test was applied when over 20% of the expected counts were below 5. *P* value less than 0.05 in two tails was considered statistically significant.

## Results

Among 178 patients determined as *H. pylori* positive by Urea breath test (UBT), successful bacterial cultures were obtained for 163 (91.57%) patients.

### Characteristics of the study population

The base information of the 163 participants infected with *H. pylori* was presented in Table 1. The age of these patients varied from 23 to 80 years with a mean age of 52.61 ± 11.204 years. Among the whole group male and female were 52 (31.90%) and 111 (68.10%), respectively. A large proportion of subjects did not conduct current smoking (92.02%; 150/163) and/or drinking behaviors (82.82%; 135/163). Subjects suffering from hypertension (20.86%; 34/163), Diabetes mellitus (14.72%; 24/163), and those whose BMI ≥25 (25.77%; 42/163) were in the minority. The majority of subjects received a diagnosis of AG (53.99%; 88/163). Tiny proportions of subjects were diagnosed with PUD, MALToma and GC at similar rates of 2.45%, 1.84%, and 2.45% respectively. There were 89.57% (146/163) subjects with experienced treatment and 10.43% (17/163) subjects without experienced treatment.

**Table 1:** Characteristics of the 163 patients infected with *H. pylori*.

**Characteristics**	**Patients (%)**
Mean age (years)	52.61 + 11.204
Gender	
Male	52 (31.90)
Female	111 (68.10)
Current smoking	
Yes	13 (7.98)
No	150 (92.02)
Current drinking	
Yes	28 (17.18)
No	135 (82.82)
BMI (kg/m2)	
<25	121 (74.23)
≥25	42 (25.77)
Hypertension	
Yes	34 (20.86)
No	129 (79.14)
Diabetes mellitus	
Yes	24 (14.72)
No	139 (85.28)
Endoscopic diagnosis	
Superficial gastritis	64 (39.26)
Atrophic gastritis	88 (53.99)
Peptic ulcer diseases	4 (2.45)
MALToma	3 (1.84)
Gastric cancer	4 (2.45)
History of *H.pylori* treatment	
Yes	146 (89.57)
No	17 (10.43)

### Antibiotic resistance rates in *H. pylori* isolates

**Figure 1:**
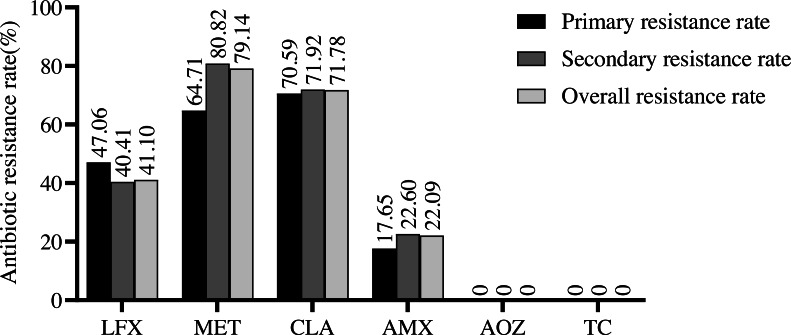
Analysis of antibiotic resistance rates in Liaoning Province. Antibiotic resistance rates among the 163 *H. pylori* strains. LFX, levofloxacin; MET, metronidazole; CLA, clarithromycin; AMX, amoxicillin; AOZ, furazolidone; TC, tetracycline.

No resistance to AOZ or TC was observed in the population. The overall resistance rates to both MET and CLA remained at about the same high level (79.14% for MET, followed by 71.78% for CLA), with AMX having the lowest rate, but still at 22.09% (Fig. 1). CLA (70.59%) owned the highest antibiotic resistance rate among the 17 isolates without *H. pylori* treatment history, along with MET (64.71%), LFX (47.06%), and AMX (17.65%). For 146 subjects once accepted *H. pylori* treatment, resistance rates in descending order were MET (80.82%), CLA (71.92%), LFX (40.41%), and AMX (22.60%), remaining in the same order as the overall resistance rates.

### Antibiotic resistance patterns in *H. pylori* isolates

Of 163 *H. pylori* isolates, eight (4.91%) were sensitive to all the six antibiotics, and mono-resistance was found with LFX (1.23%), MET (20.25%), and CLA (4.91%). 50 (30.67%) isolates were double resistant (including three to LFX+MET, 10 to CLA+LFX, 32 to CLA+MET, and five to CLA+AMX). Triple resistance was found in 42 isolates with 31 (19.02%) resistant to LFX+MET+CLA, 1 (0.61%) to LFX+CLA+AMX and 10 (6.13%) to MET+CLA+AMX. 20 (12.27%) isolates were resistant to four antibiotics including LFX, MET, CLA and AMX (Table 2). There were no *H. pylori* isolates resistant to five or six antibiotics. In the treatment-experienced population, rates of non-resistance, monoresistance, double resistance, triple resistance, and quadruple resistance were 4.79%, 26.71%, 29.45%, 26.03% and 13.01%, respectively. In the group without treatment history, the data appeared to be 5.88%, 23.53%, 41.18%, 23.53% and 5.88%.

**Table 2:** Antibiotic resistance patterns among the *H. pylori* isolates.

**Type of resistance**	**Treatment-naïve (*n* = 17)**		**Treatment-experienced (*n* = 146)**		**Overall (*n* = 163)**	
	**Number**	**Resistance rate (%)**	**Number**	**Resistance rate (%)**	**Number**	**Resistance rate (%)**
No resistance	1	5.88	7	4.79	8	4.91
Monoresistance	4	23.53	39	26.71	43	26.38
LFX	1	5.88	1	0.68	2	1.23
MET	1	5.88	32	21.92	33	20.25
CLA	2	11.76	6	4.11	8	4.91
Double resistance	7	41.18	43	29.45	50	30.67
LFX + MET	2	11.76	1	0.68	3	1.84
CLA + LFX	1	5.88	9	6.16	10	6.13
CLA + MET	3	17.65	29	19.86	32	19.63
CLA + AMX	1	5.88	4	2.74	5	3.07
Triple resistance	4	23.53	38	26.03	42	25.77
LFX + MET + CLA	3	17.65	28	19.18	31	19.02
LFX + CLA + AMX	0	0.00	1	0.68	1	0.61
MET + CLA + AMX	1	5.88	9	6.16	10	6.13
Quadruple resistance	1	5.88	19	13.01	20	12.27
LFX + MET + CLA + AMX	1	5.88	19	13.01	20	12.27

### Relationships between *H. pylori* antibiotic resistance and patient characteristics

The results of Fisher’s exact test suggested that resistance to CLA and endoscopic findings (*P* = 0.010) significantly differ (Table 3). We further explored the relationship between CLA-resistance situation and every endoscopic finding by chi-square test. A significant difference was found between CLA-resistance and MALToma (*P* = 0.021; Table 4). Another difference existed between MET-resistance and age (*P* < 0.001). No other correlation was discovered between antibiotic resistance and patient characteristics including gender, current drinking conditions, BMI, hypertension, diabetes mellitus and *H. pylori* treatment history (*P* > 0.05; Table 3).

**Table 3:** Rates of antimicrobial resistance stratified by patient characteristics were shown.

**Characteristics**	**Levofloxacin**		***P***	**Metronidazole**		***P***	**Clarithromycin**		***P***	**Amoxicillin**		***P***
	**Resistant**	**Sensitive**		**Resistant**	**Sensitive**		**Resistant**	**Sensitive**		**Resistant**	**Sensitive**	
Age (years)												
⩾50	42	64	0.620	93	13	<0.001	75	31	0.720	22	84	0.693
<50	25	32		36	21		42	15		14	43	
Gender												
Male	22	30	0.865	41	11	>0.999	34	18	0.263	13	39	0.549
Female	45	66		88	23		83	28		23	88	
Current smoking												
Yes	2	11	0.075	10	3	0.735	9	4	0.760	5	8	0.164
No	65	85		119	31		108	42		31	119	
Current drinking												
Yes	13	15	0.535	22	6	>0.999	23	5	0.249	8	20	0.452
No	54	81		107	28		94	41		28	107	
BMI (kg/m2)												
≥25	15	27	0.469	33	9	0.476	29	13	0.692	8	34	0.670
<25	52	69		96	25		88	33		28	93	
Hypertension												
Yes	13	21	0.845	29	5	0.415	20	14	0.085	6	28	0.643
No	54	75		100	29		97	32		30	99	
Diabetes mellitus												
Yes	10	14	0.415	21	3	0.293	14	10	0.141	4	20	0.601
No	57	82		108	31		103	36		32	107	
Endoscopic findings												
Superficial gastritis	24	40	0.123	48	16	0.127	45	19	0.010	14	51	0.396
Atrophic gastritis	42	46		72	16		68	20		21	67	
Peptic ulcer diseases	1	3		2	2		3	1		2	2	
MALToma	0	3		3	0		0	3		0	3	
Gastric cancer	0	4		4	0		1	3		0	4	
*H.pylori* treatment history												
Yes	59	87	0.612	118	28	0.127	105	41	>0.999	33	113	0.766
No	8	9		11	6		12	5		3	14	

**Notes.**

Significant differences were found between CLA-resistance and endoscopic findings (*P* = 0.010), and between MET-resistance and age (*P* < 0.001).

**Table 4:** Associations between endoscopic findings and antimicrobial resistance.

**Endoscopic findings**	**Levofloxacin**		***P***	**Metronidazole**		***P***	**Clarithromycin**		***P***	**Amoxicillin**		***P***
	**Resistant**	**Sensitive**		**Resistant**	**Sensitive**		**Resistant**	**Sensitive**		**Resistant**	**Sensitive**	
Superficial gastritis												
Yes	24	40	0.516	48	16	0.327	45	19	0.859	13	51	0.703
No	43	56		81	18		72	27		23	76	
Atrophic gastritis												
Yes	42	46	0.079	72	16	0.440	68	20	0.116	21	67	0.576
No	25	50		57	18		49	26		15	60	
Peptic ulcer diseases												
Yes	1	3	0.644	2	2	0.192	3	1	>0.999	2	2	0.212
No	66	93		127	32		114	45		34	125	
MALToma												
Yes	0	3	0.269	3	0	>0.999	0	3	0.021	0	3	>0.999
No	67	93		126	34		117	43		36	124	
Gastric cancer												
Yes	0	4	0.144	4	0	0.581	1	3	0.068	0	4	0.577
No	67	92		125	34		116	43		36	123	

**Notes.**

Further significant difference was found between CLA-resistance and MALToma (*P* = 0.021).

## Discussion

*H. pylori* antibiotic resistance is a major threat to affect the success of current therapies and regional difference has been an ordinary issue in the antibiotic resistance to *H. pylori*. This issue could be alleviated by antimicrobial susceptibility testing for *H. pylori*, but with harsh growth environment requirements, operational safety and economic benefits and risks to be further evaluated, so this testing method almost remains unavailable in clinical practice (Jiao, Wang & Ma, 2021; Jones et al., 2017). Under this circumstance, profiling regional antibiotic resistance patterns and specific characteristics is an extremely important measure for guiding the choice of therapeutic regimens. It is urgent to supervise respective antibiotic resistance patterns in different areas to solve the problem of indiscriminate and excessive use of antibiotics.

The resistance rate to antibiotics of *H. pylori* in China has been increasing with an upward trend during several years. From two studies, primary resistance rates to LFX, MET, CLA and AMX were all much higher than 5 years ago (Chen et al., 2022; Hu, Zhu & Lu, 2017) , as evidence of the difficult eradication today. The data about antimicrobial resistance in Liaoning were the first to be reported and revealed a situation that the overall resistance rates to LFX, MET, CLA, and AMX were higher than those in China (Chen et al., 2022) , especially for CLA (71.78% further higher than 30%), except for TC and AOZ with undetected results. These results were consistent with the conclusion by Liu et al. (2019). Such different prevalence might be correlated well with urbanization level, socioeconomic status, sanitary level and so on (Hooi et al., 2017). We found that both primary and overall antibiotic resistance rates to CLA (70.59% and 71.78%, respectively) were much higher than those in China (34% and 30%, respectively) (Chen et al., 2022) , possibly because the cold weather in north areas could cause colds and flu, and macrolides are widely prescribed for respiratory infections (Megraud et al., 2021). CLA is still widely used in many regions as an antibiotic in the standard triple therapy for *H. pylori* eradication (Fallone et al., 2016). It was recommended to avoid the usage of CLA if the local resistance to CLA was over 15% (Malfertheiner et al., 2017; Mascellino et al., 2017). Therefore, in the studied region, regimens including CLA was not appropriate for empirical treatment unless the prior antibiotic susceptibility test revealed a sensitive result with CLA. For AMX, all the AMX-resistant patients were multi-resistant. We found the primary AMX-resistant rate in our research (17.65%) is relatively higher than those in China (3.1%) (Hooi et al., 2017) or other places in China, such as Zhang et al. (2015b) in Beijing (4.4%) between 2013 and 2014 and Jiang et al. (2021) in Nanjing (1.83%). Nonetheless, the AMX-resistance rate was still the lowest among the four antimicrobials in this study. Such phenomena revealed less utilization of AMX in this area. AMX-based regimens might have the potential to be adopted as an empirical clinical treatment prescription in the future. MET-resistance rates differ all over the world. In western countries, it gained improvements after regulated uses, such as 16%, 25% and 27% respectively in Austria (Zollner-Schwetz et al., 2016), Portugal (Lopo et al., 2018) and Spain (Macias-Garcia et al., 2017). Meanwhile, in China, the use of inexpensive, easily obtained prescription medicine is easier to develop resistance (70%) (Chen et al., 2022). Within China itself, compared to other areas (Yu et al., 2019; Zhang et al., 2015b), the present study reported a high overall MET-resistance rate (79.14%). Though the typical empirical regimen(proton pump inhibitor, MET, CLA, and AMX) with increased dosage of MET or AMX might be an alternative to partly overcome the resistance to MET (Chen et al., 2019; Liang et al., 2013; Zhang et al., 2015a) , increased incidence of adverse side effects such as vomiting, nausea and rashes require consideration (Graham & Lee, 2015). Therefore, when prior susceptibility testing is not available, we suggest empirical treatment regimens containing MET be preferably abandoned. Resistance rate to LFX was up high to 41.10% in this study, higher than in China (31%), but almost similar to those in Shanghai (40.7%) (Yu et al., 2019) and Shenzhen (39.8%) (Lyu et al., 2020). Recently, LFX has been widely applied in *H. pylori* eradication treatment for the high CLA- and MET-resistance. A research discovered LFX-resistance were significant different between women and men (40.5% *versus* 21.5%), probably relating to the extent of use for urinary tract infections (Boyanova et al., 2012). We also found that the resistance to LFX presented a higher rate in female patients (27.61%) than that in male patients (13.50%). Such results suggested that we should reduce the frequency of LFX application among women population. Resistance to AOZ or TC was not detected in this study. Similar phenomenon has been observed in a population living in Sichuan Province, where the primary resistance rate of AOZ or TC was 0.8% (Tang et al., 2020). TC is not a common prescription in China, which means that semisynthetic TC derivatives such as minocycline might replace TC to gain good efficiency. Further randomized controlled trials need to be conducted.

In terms of resistance patterns, among strains with no antibiotic resistance, treatment-naïve strains accounted for a larger proportion. In other resistance patterns, treatment-experienced group was the majority. Dual resistance was an exception, treatment-naïve population (41.18%) were more frequently found than the other(29.45%). Dual resistance occupied the largest ratio (30.67%) of resistance patterns. Isolates exhibiting simultaneous resistance to CLA and MET had the largest prevalence (19.63%), followed by CLA+LFX(6.13%). Dual antibiotic resistance rate to CLA and MET from an Iran study (*n* = 12, 12%) were similar to ours and demonstrated the combination of those two antibiotics were not recommended as the first-line treatment (Hamidi et al., 2020). Based on our data, the frequency of triple antibiotic resistance was 25.77%, with LFX+MET+CLA predominating (*n* = 31, 19.02%). This might result in the failure eradication in this region and indicate advance drug susceptibility tests are required for the use of these drugs.

Some factors were investigated to influence antibiotic resistance profiles in the present study. A significant relationship was found between CLA-resistance and endoscopic diagnosis (*P* = 0.010), we further analysed that MALToma (*P* = 0.021) was associated with resistance to CLA (Table 4). It has been reported that 60–80% of MALToma patients with *H. pylori* would be in remission after eliminating the bacteria (Nakamura & Matsumoto, 2013), and long-term CLA use or the immunomodulatory drug lenalidomide also work well (Raderer & Kiesewetter, 2020). An Australia study detected MALToma patients with *H. pylori* from 1997 to 2014 and concluded CLA-resistance rate was as low as 15%, which supported eradication regimens containing CLA (Bilgilier et al., 2016). However, the present high CLA-resistance rate and its close association with MALToma suggest us antimicrobial susceptibility tests should be required among those patients to select the appropriate antimicrobial eradication therapies or refined immunomodulatory strategies. We found a significant difference between MET resistance and age (*P* < 0.001). MET is a high prescription rate medicine (Ghotaslou et al., 2018). Choe et al. (2022) concluded that previous MET exposure caused a significant reduction in eradication rate of bismuth quadruple therapy. Another study (Lee et al., 2020) calculated that the odds ratio of past MET use leading to eradication failure was 3.468. Therefore, MET resistance might be related with an increased exposure of drugs. Antibiotics should be chosen more cautiously among patients of all ages. Other factors including gender, BMI, hypertension, diabetes mellitus and so on had no significant relationship with antibiotic resistance. The results of the above data might be due to the tiny sample size, which required to be further explored by expansion of the sample size.

Our study also had limitations. There were limited primary treatment subjects in this study. This is because in clinical practice, antibiotic sensitivity tests are usually applied to patients who have failed eradication several times. Patients without previous treatment history tend to choose empirical treatment regimen, so it is difficult to collect samples from first-treatment subjects, which is also a real problem. But we still offered an updated profile of *H. pylori* infectious patients in Liaoning. Researchers could carry this study continuously, by combining multiple hospitals and expanding the sample size, especially the number of subjects without experienced treatment history, and to explore a more stable antibiotic resistance profile in Liaoning. It is vital for clinical physicians to modify prescriptions, prevent unnecessary antibiotic consumption and evaluate the effectiveness of empirical treatment in time to improve eradication rate.

## Conclusions

In conclusion, among this north China population, the primary resistance rates of LFX, MET, CLA, and AMX were relatively high and different in comparison with the overall or regional resistant data in China. To achieve good success rates, prior antimicrobial resistant tests are suggested before antibiotic prescription in *H. pylori* eradication to achieve good success rates and avoid severe antibiotic resistance.

##  Supplemental Information

10.7717/peerj.15268/supp-1 Data S1 Raw data Antibiotic resistance rates and population characteristics, such as age, gender, current smoking status, current drinking conditions, BMI, hypertension, diabetes mellitus, endoscopic diagnosis and H. pylori treatment history.

## Additional Information and Declarations

### Data Availability

The following information was supplied regarding data availability: The raw data is available as Supplemental File.
